# Association Between HIV Infection and Cancer Stage at Presentation at the Uganda Cancer Institute

**DOI:** 10.1200/JGO.17.00005

**Published:** 2017-10-16

**Authors:** Manoj P. Menon, Anna Coghill, Innocent O. Mutyaba, Warren T. Phipps, Fred M. Okuku, John M. Harlan, Jackson Orem, Corey Casper

**Affiliations:** **Manoj P. Menon**, **Anna Coghill**, **Warren T. Phipps**, and **Corey Casper**, Fred Hutchinson Cancer Research Center; **Manoj P. Menon**, **Warren T. Phipps**, **John M. Harlan**, and **Corey Casper**, University of Washington, Seattle, WA; and **Innocent O. Mutyaba**, **Fred M. Okuku**, and **Jackson Orem**, Uganda Cancer Institute, Kampala, Uganda.

## Abstract

**Purpose:**

The HIV epidemic has contributed to the increasing incidence of cancer in sub-Saharan Africa, where most patients with cancer present at an advanced stage. However, improved access to HIV care and treatment centers in sub-Saharan Africa may facilitate earlier diagnosis of cancer among patients who are HIV positive. To test this hypothesis, we characterized the stage of cancer and evaluated the factors associated with advanced stage at presentation among patients in Uganda.

**Methods:**

We conducted a retrospective analysis of adult patients with any of four specific cancers who presented for care in Kampala, Uganda, between 2003 and 2010. Demographic, clinical, and laboratory data were abstracted from the medical record, together with the outcome measure of advanced stage of disease (clinical stage III or IV). We identified measures for inclusion in a multivariate logistic regression model.

**Results:**

We analyzed 731 patients with both AIDS-defining cancers (cervical [43.1%], and non-Hodgkin lymphoma [18.3%]), and non–AIDS-defining cancers (breast [30.0%] and Hodgkin lymphoma [8.6%]). Nearly 80% of all patients presented at an advanced stage and 37% had HIV infection. More than 90% of patients were symptomatic and the median duration of symptoms before presentation was 5 months. In the multivariate model, HIV-positive patients were less likely to present at an advanced stage as were patients with higher hemoglobin and fewer symptoms.

**Conclusion:**

Patients with limited access to primary care may present with advanced cancer because of a delay in diagnosis. However, patients with HIV now have better access to clinical care. Use of this growing infrastructure to increase cancer screening and referral is promising and deserves continued support, because the prognosis of HIV-positive patients with advanced cancer is characterized by poor survival globally.

## INTRODUCTION

The incidence of cancer is increasing globally, with nearly 14 million new cases diagnosed in 2012.^1^ The burden of cancer is growing in sub-Saharan Africa (SSA), where an estimated 766,000 incident cancer cases and 587,000 cancer deaths are projected to occur in 2020, an increase of approximately 40% over 2008.^1^ This problem is particularly noteworthy given the prevalence of concomitant HIV infection in SSA because infection with HIV is associated with an increased risk of a variety of malignancies, likely in part because of systemic immunosuppression.^2^ Although the increased risk is most pronounced for malignancies caused by oncogenic infections (eg, Kaposi’s sarcoma; anogenital cancers; or certain subtypes of non-Hodgkin lymphoma [NHL] including Burkitt’s lymphoma, primary CNS lymphoma, and primary effusion lymphoma), the risk persists for many cancers.^3,4^ Fortunately, the incidence of many cancers among HIV-infected individuals is declining,^5,6^ possibly because of an effective combination antiretroviral therapy (cART) that assists in immune reconstitution and the prevention of severe immunosuppression.^7^ However, HIV-infected patients are still typically diagnosed at a later stage, with worse outcomes than uninfected patients with cancer.^8^

In low- and middle-income countries, including those in SSA, the majority of patients with cancer, independent of HIV status, present to care at an advanced stage. Although there is clearly variability among countries in SSA, one factor responsible for the late stage at presentation is secondary to a limited health care infrastructure that precludes access to timely clinical care and the resulting lack of medical surveillance.^9-12^ Similarly, in the United States, in a study linking cancer registry data with registry data from HIV and AIDS and solid-organ transplant populations, both of which are immunosuppressed groups, it was observed that HIV-infected patients were more likely to present at an advanced stage of lung, breast, and prostate cancer than were immunocompetent patients. In contrast, solid-organ transplant patients were more likely to present with early-stage cancer than were immunocompetent patients, suggesting a potential role of medical surveillance and increased vigilance among transplant recipients.^13^

In large part because of funding from the Global Fund for AIDS, TB, and Malaria and the US President’s Emergency Fund for AIDS Relief (PEPFAR), access to HIV care and treatment, including the availability of cART, has increased dramatically in SSA.^14^ As such, HIV-infected patients now likely have improved access to clinical care, as well as access to cART, and therefore may benefit from stricter medical follow-up. The benefit of such vertical programmatic efforts on other health outcomes, including cancer, is uncertain. A recent retrospective longitudinal study in Uganda did not reveal any significant changes in non-HIV service use despite PEPFAR investments in strengthening health systems; however, data from other countries have been encouraging.^15^ Therefore, we hypothesize that HIV-infected patients with cancer present to care at an earlier stage than do their uninfected counterparts and that by using the HIV care infrastructure, clinical outcomes may be improved. Here we describe the association between HIV status, and other patient characteristics, and advanced stage of cancer at diagnosis among patients presenting for care in Uganda.

## METHODS

We conducted an analysis of adult (> 18 years of age) residents of Kyadondo County (Uganda) who were diagnosed with breast cancer, cervical cancer, NHL, or Hodgkin lymphoma (HL) between 2003 and 2010 as part of a retrospective cohort study described previously.^10^ Cases were identified from the Kampala Cancer Registry, a population-based cancer registry covering the capital city, Kampala, and the surrounding peri-urban regions. Data from the Kampala Cancer Registry were reconciled with clinical data from a national teaching hospital in Kampala, Uganda (Mulago Hospital) and the adjacent Uganda Cancer Institute (UCI), the nation’s only cancer center. The four malignancies analyzed here represent cancers typically associated with an infection (ie, cervical cancer and human papilloma virus, certain subtypes of NHL and Epstein-Barr virus, HL and Epstein-Barr virus) and a cancer with no known association with infection (ie, breast cancer). In addition, we deliberately included both AIDS-defining cancers (ie, cervical and NHL) and non–AIDS-defining cancers (ie, HL and breast). Finally, we assessed cancers that were a particular burden in Uganda; cervical and breast cancer represent the most common and the second most common cause of cancer and cancer-related deaths among women in Uganda, respectively.^1^ Only patients with a new diagnosis of cancer were included; patients with relapsed or refractory disease were excluded.

We abstracted demographic and clinical data from the medical record. Clinical data included a review of symptoms, duration of symptoms, medical history (including medications and comorbid conditions), and physical examination. Separate composite measures of the number of symptoms (ie, symptom score) and coexisting medical illness (ie, comorbidity index) were created; each individual symptom or illness was given the same weight. Laboratory data included blood counts and metabolic measurements when available. Anemia was defined as a hemoglobin level < 11 g/dL per the WHO definition. The stage at presentation, assessed typically via an abdominal ultrasound and/or a chest radiograph, was dichotomized as nonadvanced or advanced stage and was categorized as per standard clinical staging systems. For both HL and NHL, nonadvanced disease included either the involvement of a single lymph node region (stage I) or the presence of two or more lymph node regions on the same side of the diaphragm (stage II). Advanced disease was characterized by involvement of lymph node regions on both sides of the diaphragm (stage III) or by diffuse disease (stage IV).^16-18^ For cervical cancer, nonadvanced disease was limited to invasion beyond the uterus but not to the pelvic wall or lower third of the vagina; extension to the lower third of the vagina or the presence of any nodal disease was characterized as advanced stage as per the International Federation of Gynecology and Obstetrics.^19^ Breast cancer staging was per the American Joint Commission on Cancer. The presence of any nodal involvement, with the exception of ipsilateral axillary nodes in the setting of a small primary tumor (ie, < 20 mm), was characterized as advanced disease. In addition, any tumor involvement with direct extension to the chest wall or skin, independent of nodal status, was characterized as advanced disease.^20^ In the event that clinical stage was not recorded in the medical record, a study physician at the UCI reviewed the medical record and assigned a clinical stage.

HIV status was ascertained by either the results of HIV antibody testing, documentation of care at a local HIV treatment facility, or documentation of HIV status in the clinical notes.

The primary outcome measure was advanced stage of disease (ie, stage III or IV) at presentation. Using logistic regression models, we assessed whether infection with HIV, as well as whether other demographic, clinical, and laboratory measurements, were associated with stage. Variables with a *P* value < .20 in the bivariate model were included in the multivariate logistic regression analysis.^21^ These models were used for each individual malignancy as well as for the total patient sample overall.

The study was approved by the Makerere University College of Health Sciences Research Ethics Committee (Kampala, Uganda) and by the Fred Hutchinson Cancer Research Center’s institutional review board (Seattle, WA).

## RESULTS

A total of 731 patients were included in this analysis, including 315 women with cervical cancer, 219 patients with breast cancer, 134 patients with NHL, and 63 patients with HL. The median age of all patients was 43 years (18 to 86 years), with little variation by cancer type (Table 1).

**Table 1:**
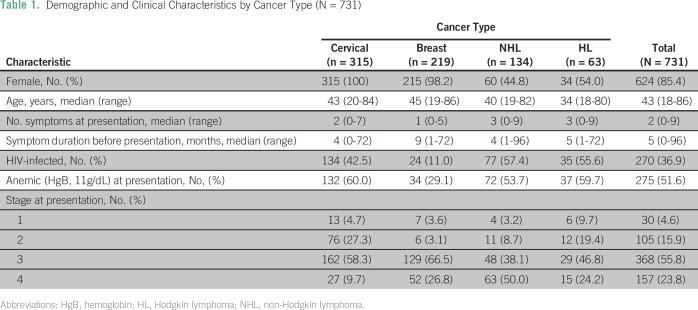
Demographic and Clinical Characteristics by Cancer Type (N = 731)

More than one half of patients with either NHL (77 [57.5%]) or HL (35 [55.6%]) were HIV infected, whereas 42.5% of women with cervical cancer were HIV infected (n = 134); a lower percentage of patients with breast cancer were HIV infected (24 [11.0%]) compared with patients with the other cancers studied. Overall, 525 (79.6%) presented at an advanced stage of disease. Approximately 70% of women with cervical cancer (199 [68%]) and patients with HL (44 [71%]) presented at an advanced stage, whereas nearly 90% of patients with breast cancer (181 [93.3%]) and NHL (111 [88.1%]) were diagnosed with advanced disease at presentation (Table 1).

At the time of initial presentation, patients presented with a median of two symptoms (range, zero to nine symptoms); these symptoms were present for a median of 5 months before presentation (range, 0 to 96 months). Although women with breast cancer presented with a median of one symptom at presentation, the median duration was 9 months (range, 1 to 72 months) before presentation (Table 1). More than one half of the patients with NHL (72 [53.7%]), HL (37 [59.7%]), and cervical cancer (132 [60.0%]) were anemic at presentation; a slightly lower percentage of patients with breast cancer (29.1% [34 patients]) were anemic. With the exception of tuberculosis, which was a coexisting illness among 14.9% of patients (n = 20) and 23.8% of patients (n = 15) with NHL and HL, respectively, other comorbidities were uncommon.

The association of the various demographic and clinical characteristics with advanced stage at diagnosis differed according to cancer type in our study cohort (Table 2). Among patients with breast cancer, an increased symptom score and a longer duration of symptoms before presentation were associated with advanced stage of disease in unadjusted analyses, with symptom score remaining significantly associated with advanced disease in adjusted models (odds ratio [OR], 19.5; 95% CI, 1.3 to 293.2). In the unadjusted analyses among women with cervical cancer, HIV-infected patients were more likely than were HIV-uninfected patients to present at an early stage; other factors included an older age, an increased symptom score, an increased comorbidity index, and lower hemoglobin, with only hemoglobin at the time of presentation remaining associated with advanced stage in adjusted models (OR, 0.81; 95% CI, 0.71 to 0.93). An increased symptom score and lower hemoglobin were associated with NHL in the adjusted analysis; however, neither of these factors was associated with advanced stage of disease. Among patients with HL, of the factors significant in the unadjusted analysis (increased symptom score, additional comorbidities, lower hemoglobin, and HIV infection), only HIV infection was significantly associated with the likelihood of presenting with advanced-stage disease.

**Table 2:**
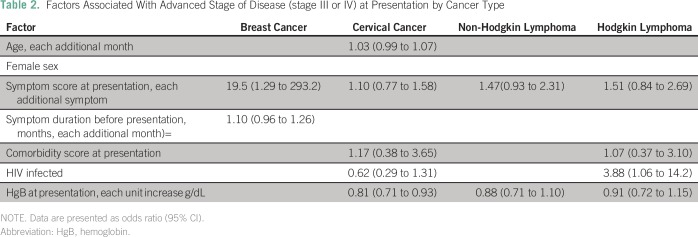
Factors Associated With Advanced Stage of Disease (stage III or IV) at Presentation by Cancer Type

**Fig 1:**
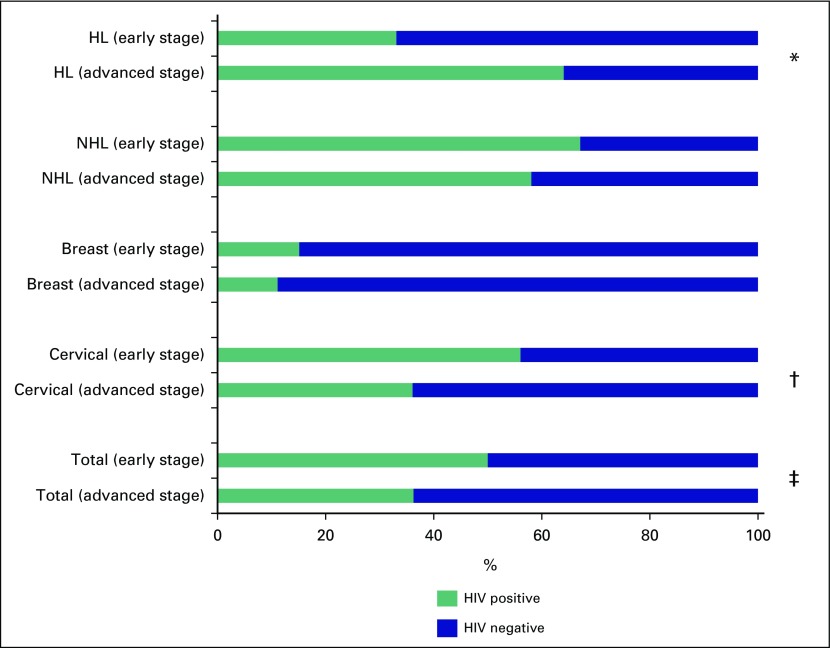
Prevalence of HIV infection by cancer type and stage. (*) OR, 3.5; 95%CI, 1.10 to 11.1. (†) OR, 0.4; 95% CI, 0.26 to 0.73. (‡) OR, 0.5; 95% CI, 0.35 to 0.74. HL, Hodgkin lymphoma; NHL, non-Hodgkin lymphoma; OR, odds ratio.

Among all patients with cancer, HIV infection, older age, male sex, increased number of symptoms, longer symptom duration, and anemia were associated with advanced disease in the unadjusted analyses (Table 3). After adjusting for the other covariates, HIV-infected patients were more likely to present at an earlier stage for the entire sample (OR, 0.53; 95% CI, 0.30 to 0.94); however, this was driven largely by the relationship between HIV infection and advanced cervical cancer (OR, 0.62; Table 3 and Fig 1). In addition, each additional symptom (OR, 1.54; 95% CI, 1.18 to 2.03) and a lower hemoglobin (OR, 0.89; 95% CI, 0.80 to 0.99) were significantly associated with an increased odds of presenting at an advanced stage in the multivariate model (Table 3).

**Table 3:**
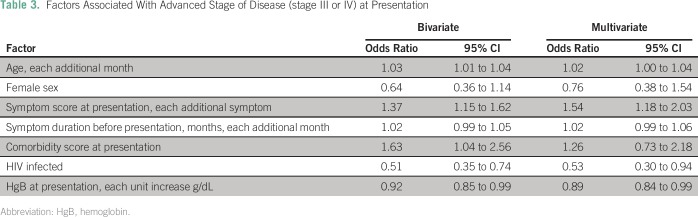
Factors Associated With Advanced Stage of Disease (stage III or IV) at Presentation

## DISCUSSION

Patients with cancer in resource-poor settings often have limited access to primary care and may therefore delay presenting to cancer specialty care. In our cohort, nearly 80% of patients presented to care at an advanced stage. A host of factors, including both patient specific (ie, lack of patient awareness, perceived costs, and preference for traditional healers) and system related (ie, a weak health care infrastructure resulting in a lack of trained health care workers and diagnostics), have been documented previously as possibly responsible for the corresponding delay to care.^22,23^ Multiple studies have documented the negative impact of delays to diagnosis, including increased mortality.^24-28^ In a study of nearly 3,000 women with breast cancer, delays to care, defined as the presence of symptoms for ≥ 12 weeks before presentation were associated with inferior survival, an association caused by the relationship between advanced stage and delay.^26^

In our entire sample, nearly 80% of patients presented at an advanced stage, including > 90% of women with breast cancer and nearly 70% of women with cervical cancer. Largely because of breast and cervical cancer screening, < 20% of women with breast cancer and < 10% of women with cervical cancer present with distant disease in the United States.^29-31^ Accordingly, in the United States, the 5-year survival rate ranges from 58% to 93% for early-stage cervical cancer compared with 15% to 35% for advanced-stage disease.^20^ Among patients with breast cancer, the 5-year survival for stage I disease approaches 100%, compared with 22% for patients with stage IV disease.^20^ Similarly, among our sample, nearly 90% of patients with NHL and 70% of patients with HL presented with advanced disease compared with approximately 50% and 40% of patients with NHL and HL, respectively, in the United States.^29,32^ Both the International Prognostic Index and the International Prognostic Score for NHL and HL document the adverse effect of advanced-stage disease.^33,34^ Although not validated prospectively, these relationships almost certainly exist in resource-limited areas as well. As such, efforts to identify patients at an earlier stage, when prognosis and treatment options are improved, are clearly warranted.

Among our sample, HIV-infected patients presented at an earlier stage than did their uninfected counterparts, a likely benefit of more timely clinical access and engagement. As such, one potential strategy to improve the early detection of cancer, and therefore downstage patients in resource-limited regions, is to strengthen the health care infrastructure by leveraging the strengths of vertical health programs via a diagonal approach.^35,36^ Given the high prevalence of concomitant HIV infection among patients at the UCI (eg, nearly 40% in our sample), efforts to integrate HIV care and treatment programs into cancer screening and early-detection programs would be beneficial. Multiple initiatives, including the Global Fund, PEPFAR, and the World Bank Multicountry AIDS Program, have dramatically increased the scale-up of HIV-AIDS service delivery in SSA. Although these efforts have clearly had positive effects on reducing the incidence of HIV, a criticism of these vertical initiatives is that they may weaken the overall health system by increasing demand and decreasing the workforce via worker burnout.^14,37,38^

In SSA, efforts to integrate health promotion activities, including family planning and making available safe water and childhood vaccinations, have been successful using the HIV care and treatment platform.^39,40^ Indeed, in a study of patients in HIV care and treatment centers in Ethiopia, the integration of basic care services aimed at improving sanitation and hygiene among HIV-infected patients receiving cART improved health outcomes (eg, lower rates of illness, less health facility visits).^40^ It is especially important to diagnose and treat HIV-infected patients early, given their increased cancer-specific mortality compared with that of uninfected individuals.^8,41^ Multiple studies have documented the increased mortality associated with cancer among HIV-infected patients both in resource-abundant and in resource-limited regions, likely secondary to both HIV-induced immunosuppression and the decreased likelihood of receiving cancer-directed therapy.^10,42-45^

In addition to HIV infection, patients in our retrospective cohort with a higher symptom score (ie, a greater number of symptoms) and those with lower hemoglobin, both possibly suggestive of a greater disease burden, were more likely to present at an advanced stage. Although a patient’s functional status (eg, Eastern Cooperative Oncology Group performance status) has prognostic usefulness, it was not recorded routinely in these medical records.^46,47^ However, a recent cross-sectional study among patients with cancer in Botswana noted that the symptom burden, as measured by the Memorial Symptoms Assessment Scale–Short Form, was significantly associated (*P* < .01) with the patient’s Eastern Cooperative Oncology Group performance status.^48^ It is unknown, however, whether the prognosis associated with increased symptoms or poor performance status is reflective of the biology of the disease or whether such symptoms preclude the use of cancer-directed therapy. Similarly, the presence of anemia among patients with terminal cancer has been shown previously to be associated with poor prognosis and early mortality.^49-51^ However, the direction of these associations is not clear. Although the association between anemia and poor health outcomes among patients with cancer is well documented, it is uncertain whether anemia is a marker for more aggressive or refractory disease, or whether anemia limits or affects treatment options (ie, delaying or deferring chemotherapy). Regardless, symptomatic patients would benefit from early clinical care; however, the health care infrastructure in SSA often precludes such clinical engagement.

Given the recent increase in HIV care and treatment centers in SSA, patients with HIV likely have improved access to clinical care. Although much of the integration of HIV care has been focused either on other infectious diseases or on maternal health, recent efforts have recognized the increasing burden of noncommunicable diseases in resource-limited regions. Because cervical cancer remains a leading cause of morbidity and mortality in SSA, with an increased incidence among HIV-infected women, limited efforts have begun to integrate cervical cancer screening programs within HIV treatment platforms. Using PEPFAR support, colleagues in Mozambique implemented a 1-year cervical cancer screening pilot program, via visual inspection with acetic acid, in four health facilities that provide cART. Although not performed routinely in SSA, visual inspection with acetic acid in this pilot study was positive in 380 of the 4,651 women screened (8%), the majority of whom had never been screened previously. Nine months after implementation, > 95% of women requiring treatment via cryotherapy received therapy on the day of screening, demonstrating a benefit of screening in early diagnosis and treatment.^52^ Using a computer simulation model, researchers estimated that cervical cancer screening at cART initiation would prevent one cervical cancer–related death for every 262 HIV-positive women screened in Cameroon.^53^ Although the number needed to screen in that analysis was higher than in an analysis of the United Kingdom National Cervical Screening Program, in which cervical cancer screening was found to prevent the death of one in 65 screened woman, it compares favorably to the screening benefit of mammography.^54,55^ Whereas the cost effectiveness of cervical cancer screening has been documented among women in resource-limited settings and among HIV-infected women in the United States, additional data regarding the cost effectiveness of cervical cancer screening among HIV-infected women in resource-limited regions are warranted.^56,57^

Although future studies are necessary to evaluate cancer-specific predictors of advanced disease stage, leveraging the HIV care and treatment infrastructure to increase cancer screening and referral, especially with regard to cervical cancer, is a promising and likely cost-effective method to diagnose cancer at an earlier stage. Because the prognosis of HIV-infected patients with advanced-stage cancer is characterized by poor survival, even in resource-abundant regions, such integrative efforts deserve continued support.
